# A global expert elicitation on present-day human–fire interactions

**DOI:** 10.1098/rstb.2023.0463

**Published:** 2025-04-17

**Authors:** Cathy Smith, Ol Perkins, Jayalaxshmi Mistry, Bibiana A. Bilbao, Rebecca Bliege Bird, Amy Cardinal Christianson, Kayla Maria de Freitas, Wolfram Dressler, Erle C. Ellis, Ludivine Eloy, Cynthia Fowler, Simon Haberle, Jed O. Kaplan, Paul Laris, James Millington, Claudia Monzón-Alvarado

**Affiliations:** ^1^ Department of Geography, Royal Holloway University of London, Egham, UK; ^2^ Leverhulme Centre for Wildfires, Environment and Society, London, UK; ^3^ Department of Geography, King’s College London, London, UK; ^4^ Departamento de Estudios Ambientales, Universidad Simón Bolívar, Caracas, Venezuela; ^5^ ART-Dev, Univ Montpellier, CIRAD, CNRS, Univ Paul Valéry Montpellier 3, Univ Perpignan Via Domitia, Montpellier, France; ^6^ Department of Anthropology, Pennsylvania State University, University Park, PA, USA; ^7^ Indigenous Leadership Initiative, Quebec, Canada; ^8^ School of Geography, Earth and Atmospheric Sciences, University of Melbourne, Melbourne, Victoria, Australia; ^9^ Geography & Environmental Systems, University of Maryland, Baltimore, MD, USA; ^10^ Wofford College, Spartanburg, SC, USA; ^11^ CABAH, School of Culture, History & Language, The Australian National University, Canberra, Australia; ^12^ Department of Earth, Energy, and Environment, University of Calgary, Calgary, Alberta, Canada; ^13^ California State University, Long Beach, CA, USA; ^14^ CONAHCYT – ECOSUR, Lerma, Campeche, Mexico

**Keywords:** human fire use, indigenous fire ecologies, fire policies, expert elicitation, contested knowledges

## Abstract

Human fire use contributes to fire regimes and benefits societies worldwide yet is poorly understood at the global scale. We present the Global Fire Use Survey (GFUS), an effort to elicit and systematize knowledge about fire use from experts, including academics and practitioners. The GFUS data cover the stakeholders using fire, reasons for and seasonality of burning, recent trends in anthropogenic ignitions and burned area and the presence/absence and effectiveness of different policy interventions targeting fire use. The survey garnered 311 responses for regions covering over 50% of the Earth’s ice-free land, improving on the coverage of literature syntheses on fire use. Here, we analyse the data on the distribution of fire use and policy interventions. The survey suggests that the most widespread fire users are Indigenous and local people burning to meet objectives associated with small-scale livelihoods and cultural priorities, whereas burning by commercial land users, state agencies and non-governmental organizations is less widespread. Regulatory restrictions are the most common policy interventions targeting fire use but are ineffective in achieving their aims in regions with higher burned area. While community-led governance of burning is rarer, it was deemed more effective than restrictive policy interventions, particularly in regions with higher burned area.

This article is part of the theme issue ‘Novel fire regimes under climate changes and human influences: impacts, ecosystem responses and feedbacks’.

## 1. Background

Human fire use provides economic and cultural benefits to diverse people worldwide. Some fire users, including Indigenous and local Peoples, burn to meet small-scale livelihood objectives and/or for related cultural reasons (e.g. for hunting, pastoralism, cropping, fuel management or ceremony [1–3]). Larger landowners and commercial enterprises also use fire (e.g. in land clearance for industrial agriculture, forestry or cattle ranching [3]). Some state agencies and non-governmental organizations (NGOs) also conduct prescribed burning (e.g. for ecosystem or fuel management [3]).

Recent centuries have seen reductions and changes in human fire use in landscapes worldwide, driven, among other factors, by the dispossession of Indigenous peoples from their land, by agricultural intensification and state fire suppression policies [1,4]. Many landscapes are becoming more flammable through combined effects of changes in land use, fire use and climate, and societies must adapt to changing fire regimes, balancing the benefits of fire use with wildfire risk [5]. Case studies point to some of the challenges faced. In some landscapes, controlled (prescribed) fire use could help to manage fuel loads and wildfire risk, but support for this is lacking in some contexts (e.g. where colonial fire suppression policies are retained, or fire knowledge has been lost among the general public [6,7]). Where it has policy support, controlled burning may be seen as the sole prerogative of specialist agencies, but these can be strongly limited in resources and by the risk of liability if fires become uncontrolled [6]. In limited cases, there is policy support for the revival of Indigenous or local fire use where it was historically suppressed, and partnerships are emerging between agencies and Indigenous and local peoples to manage fire, incorporating local fire knowledge [8,9]. In other landscapes, controlled burning may not be desirable, and other forms of fire management, such as creating green fire breaks, may be more appropriate [10].

However, existing research on human–fire interactions at the global scale is very limited and falls into three broad categories: literature synthesis, expert elicitation and remote sensing. Recent global syntheses have distilled findings from across detailed localized case studies of human fire use and management, but this literature exhibits biases towards certain geographies and types of fire users, underrepresenting regions such as Central Africa and Western and Central Asia, and commercial fire users [1,3,11,12]. Only two studies have employed expert elicitation to examine human–fire interactions globally. One gathered knowledge from academics to assess broadscale human impacts on wildfire globally from the early Holocene to the year 2300, finding increases in human influence on fire frequency, extent and severity since 1800 [13]. Another global study, biased towards Europe, explored how state and NGO fire managers understand local fire regimes, including questions about their use of fire [14].

There have been no similar global efforts to gather expert knowledge about burning by other stakeholders; in general, the situated and practical knowledge of fire users and managers has been neglected by researchers [15]. Fire remote-sensing data, combined with proxies for human influence, like population, have also been used to make inferences about human influences on fire regimes (e.g. [16]). Yet, until recently, remote-sensing products were too coarse in scale to detect many anthropogenic fires, which are generally smaller than lightning-caused fires. Recent advances in fine spatial-resolution remote sensing should enable greater understanding of the role of anthropogenic fire in global fire regimes [17]. Yet, even with these advances, separating different types of anthropogenic fires and distinguishing these from lightning fire cannot be done without process-based understanding at the landscape scale: the challenge of ‘putting people into pixels’ remains [18].

Several consequences flow from this fragmented understanding of human–fire interactions. First, the role of human fire use in shaping fire regimes and how this relates to biophysical drivers of fire is unclear [19,20]. Second, global fire assessments tend to focus on wildfire and on outcomes like greenhouse gas emissions and risk to property and health, while the economic and cultural benefits of fire use are rarely considered (e.g. [21,22]). Finally, there is a risk that practices and policies are transferred between regions without consideration of whether they are suited to different contexts [8,23,24]. For example, based on experience from Northern Australia, development organizations are promoting early dry season burning for climate change mitigation in African savannahs, without always accounting for existing local fire use practices and governance [25–27].

Here we present the Global Fire Use Survey (GFUS), which systematically surveyed those involved in fire management across the globe, including practitioners and policymakers, as well as researchers studying human fire use. Given the paucity of existing data at this scale, the aims of the survey were exploratory—to gather a range of basic information about human fire use, including the stakeholders using fire, reasons for burning, seasonality of burning and policy interventions targeting fire use, and to make this data publicly available for others to learn from (including through a web application—see data availability statement). For example, the data could help to share understanding and promote dialogue between fire managers and/or policymakers from different regions. In this paper, we present the survey methodology, and initial analysis of some of the survey data, focusing on the spatial distribution of fire use and policy interventions targeting fire use. In the discussion we reflect on what the data reveal about global adaptation to changing fire regimes.

## 2. Methods

### (a) Survey design and participant recruitment

The GFUS was initially conceived of and designed over a year of monthly meetings by an interdisciplinary, international, team of nine researchers. In 2022, the draft survey was piloted with six researchers with expertise in human fire use. The pilot participants were asked to provide feedback for each survey question and broader comments on survey design, structure and language. They were then invited to online meetings to discuss their feedback. The pilot participants were then invited to comment on another survey draft, and subsequently invited to co-author this paper in recognition of their contribution to the survey design.

We aimed to collect data on contemporary fire use and chose to define this as the period between 1990 and 2022. We thus recruited survey participants who had been active as researchers or worked in a capacity related to fire use in their region of expertise for any period in this timeframe. People with the knowledge required to participate were wide-ranging in background and included researchers, land managers, policymakers and people using fire as part of their livelihoods. They might have subsistence livelihoods, be self-employed or work for universities, state agencies, NGOs or businesses. To enable wider participation, the survey was translated from English into French, Spanish and Portuguese by native speakers who were also fire researchers, enabling them to translate specialist terms accurately. In practice it was difficult, both for logistical (e.g. language, lack of internet access, lack of familiarity of filling out surveys) and ethical reasons (e.g. lack of direct benefits to participation) to recruit many Indigenous fire users as survey respondents. We expand on how this limits the dataset in the discussion.

Potential survey participants were initially identified via literature. We emailed invitations to participate to the authors of sources in two databases of literature on human–fire interactions, the Livelihood Fire Database (LIFE) [28], and the Database of Anthropogenic Fire Impacts (DAFI) [29]. The research team also shared the survey within their networks, and it was advertised on social media via the research team’s institutions. All those invited to participate were asked to recommend other possible participants. After the survey had collected responses for a month, we targeted additional searches for participants in regions without existing responses, through Google Scholar literature searches and by searching online for agencies and NGOs working with fire in the region.

We collected survey responses from December 2022 to June 2023. Participation was voluntary, and respondents were offered the opportunity to enter a prize draw to win a blanket designed by an Indigenous artist. The survey took approximately 30 min to complete.

### (b) Survey content

Survey participants responded for a region where they had experience researching or working with/on fire. They could answer for multiple regions by completing the survey multiple times or by indicating if they felt their answers applied equally to another region. The survey regions (https://firemap.terraces.hku.hk/) were determined by combining a large-scale map of global biomes [30] with national boundaries, e.g. the temperate coniferous forests of China were one survey region, and the tropical and sub-tropical moist broadleaf forests of China were another. We delimited the regions on this basis because we expected fire governance and land use to vary in accordance with local, regional and national legislation and economic conditions, and fire behaviour and land use to vary strongly by biome. We also expected that respondents might find it difficult to generalize across national borders since expert knowledge is often specific to national or regional political entities.

Where biomes were not geographically continuous within a country, we treated fragments of the same biome as one region. For tropical savannas, we further subdivided the regions into arid savannas where precipitation constrains woody cover, and mesic savannas, where fire and other disturbances are responsible for the coexistence of trees and grasses, using approximately 650 mm as a precipitation cut-off for African savannas [31] and approximately 1000 mm for savannas elsewhere globally [32]. For boreal forests, in Europe and Asia, we subdivided the (initially very large) regions further to the level of ecoregions [30], and in Canada, into two regions—East and West. In Australia, we examined biomes within state/territory boundaries because we knew them to have different fire policies.

We designed and administered the survey using the Qualtrics online software. There were four blocks of questions (fully detailed in electronic supplementary material, S1). The first focused on the stakeholder groups using fire, reasons for burning and months in which specific fire uses occur. Here, we considered three stakeholder groups separately: (i) small-scale livelihood and/or cultural (hereafter SSLC) fire users—Indigenous peoples and others whose livelihoods rely on family labour or labour exchange with other households; (ii) commercial fire users—large landowners and companies employing workers; (iii) state/NGO fire users—government agency or NGO land managers. The second block focused on the human fire regime, including fire use by all stakeholders. This included questions about seasonal variation in burned area, fire ignitions and risk of escaped fire, and about trends in burned area and ignitions since 1990. The third focused on fire governance, with questions about the amount of fire suppression by agencies, policy interventions affecting fire use and the effectiveness of these. The fourth looked at the respondents’ expertise, including their professional background, research methods (if applicable) and specific years and locations for which their knowledge was deepest.

When responding to the survey questions (other than those about longer-term trends), respondents were asked to consider conditions over the past 5 years (2018−2022). To enable comparison across diverse regions globally, we prioritized collection of categorical data, comparing, for example, presence/absence of descriptively defined categories of fire user, reasons for burning or policy intervention types, or asking respondents to quantify variables such as policy effectiveness on ordinal scales (e.g. high, medium and low). Respondents could also add clarifying text with additional detail for each survey block. For every question, respondents rated their confidence in their answer on a scale from 1 to 5, being asked to consider the recency of their knowledge and its applicability across the whole region. Some questions were compulsory, while others could be answered with ‘I don’t know’ (electronic supplementary material, S1).

### (c) Survey analysis

Survey analysis for this paper largely focused on high-level questions that we expected to be answered most robustly by the survey respondents (table 1); also aspects of the data that we could compare with existing datasets. These were the presence/absence in a region of different fire user types, reasons for burning and fire policy intervention types. Data were evaluated in comparison with two literature meta-analyses—the LIFE [28] and the DAFI [29]. This evaluation is detailed in electronic supplementary material, S3. We also analysed data on the reported effectiveness of different fire policy types. When assessing ‘effectiveness’, survey respondents were asked to rate on a scale of 1–3 how successful policies were in meeting their own objectives vis-à-vis fire use (e.g. to reduce it, limit it to certain times of year, etc.).

**Table 1.** Wording of survey questions and answer categories for which we analyse data in this paper.

survey question	response options
which of the following groups use fire in the landscape in this region?	(1) people who use fire to support small-scale livelihoods and/or for cultural reasons (2) people working for commercial enterprises and/or large landowners (3) protected area managers and/or people working for state agencies
why do they use fire on the land today? (Question repeated for each fire user group present in the region)	29 categories—see electronic supplementary material, table S1.2
which of the following types of governance apply to fire use in this region?	(a) state regulations making it illegal to use fire in the landscape for any reason (b) state regulations making it illegal to use fire for certain reasons. *For example, if it is illegal to use fire for hunting but legal to use fire for agriculture* (c) regulations limiting or banning fire use within protected areas. *For example, if it is illegal to use fire for agriculture in nature reserves but legal to use fire for agriculture outside nature reserves* (d) state regulations making some or all types of fire use conditional upon certain criteria. *For example, if it is only legal to use fire if the fire user has a permit to do so, the burning takes place in certain months, or the fire user has created firebreaks* (e) economic incentives to reduce or limit fire use. *For example, if agricultural subsidies are not available for agriculture involving fire use, or if payments under payments for ecosystem services schemes are conditional upon not using fire* (f) informational or educational campaigns designed to encourage reduced or limited fire use. *For example, training to encourage changes in fire use, or information provided by radio, newspaper, television etc. intended to encourage changes in fire use* (g) local forms of governance based on traditional knowledge, intended to ensure fire use is controlled. *For example, rules set by community leaders, or verbal or written community agreements about where, when or how fire should be used*
for the types of governance you selected, do you feel they are effective in achieving their intended outcome, in terms of fire use?	(i) very effective (ii) somewhat effective (iii) not effective

For each aspect of the data, we tested for statistical differences across continents and according to the backgrounds of survey respondents. We also tested whether responses varied with the annual burned area in the underlying ecosystem (measured with 5th Global Fire Emissions Database (GFED5) data [17]). We measured effect size for continuous variables (i.e. GFED5 burned area data) with Pearson’s *r*. For categorical variables (continent, respondent background), we conducted analysis of variance tests and measured the effect size with *η*^2^, which describes the proportion of variance in a target variable explained by the independent variable [33]. In addition to these three sets of tests, we also conducted statistical tests to assess the coherence of GFUS data with the LIFE and DAFI (see electronic supplementary material, S3). As such, a Bonferroni correction was applied to *p*-values, and those less than 0.0125 were taken as significant (i.e. 0.05/4).

To increase geographical coverage, survey respondents were asked if they felt that their answers could reasonably apply to any other survey regions. As such, where a survey region lacked a specific response (i.e. a respondent choosing it as their primary region of expertise), we used data from secondary or tertiary regions indicated in other survey responses.

## 3. Results

### (a) Overview of survey responses

A total of 311 respondents completed the survey, covering 174 survey regions (electronic supplementary material, S1 and figure S2.1); 30 of these regions were covered using respondents’ secondary or tertiary nominated region. The median number of responses per region was 1, the mean 2.07 and maximum number for a single region was 20. Response rates differed significantly across continents (table 2). The highest mean response rate per region was in North America (0.981), while the lowest was in Africa (0.26). Researchers made up the highest proportion of respondents (table 2; electronic supplementary material, S2 and figure S2.3a,b): 72% identified as researchers, followed by 22% as fire managers. Together, 13% of respondents use fire within their livelihoods—whether for small-scale livelihood activities or a company. Because respondents could identify with multiple roles, 57% of respondents who identified as fire managers also identified as researchers, with lower proportions of livelihood and commercial fire users (≤36%) also identifying as researchers.

**Table 2.** Statistical differences in survey data by respondent background, across continents and with GFED5 burned area. *p*-values < 0.0125 (bolded) were taken as significant. Survey respondents perceive that fire governance loses its effectiveness with increasing burned area. Perhaps relatedly, fire use for fuel load management has no global relationship with increasing burned area. SSLC, small-scale livelihood and/or cultural fire users; NGO, non-governmental organizations; GFED5, 5th Global Fire Emissions Database.

	explanatory variable		
survey domain	difference by respondent background	difference by continent	correlation with GFED5 burned area
spatial and sectoral distribution of respondents	*η*^2^ = 0.26 (0.23, 0.27) ***p* < 0.001**, d.f. = 7 2880	*η*^2^ = 0.03 (0.01, 0.05) ***p* < 0.001**, d.f. = 5690	*r* = 0.15 (0.08, 0.23) ***p* < 0.001**, d.f. = 694
SSLC fire use: presence/absence	*η*^2^ = 0.05 (0.00, 0.07) *p* = 0.097, d.f. = 7235	*η*^2^ = 0.02 (0.00, 0.04) *p* = 0.533, d.f. = 5168	*r* = 0.21 (0.07, 0.35) ***p* = 0.005**, d.f. = 172
commercial fire use: presence/absence	*η*^2^ = 0.03 (0.00, 0.04) *p* = 0.472, d.f. = 7235	*η*^2^ = 0.08 (0.01, 0.12) *p* = 0.018, d.f. = 5168	*r* = 0.11 (−0.04, 0.25) *p* = 0.153, d.f. = 172
state/NGO fire use: presence/absence	*η*^2^ = 0.07 (0.01, 0.11) ***p* = 0.012**, d.f. = 7235	*η*^2^ = 0.13 (0.04, 0.19) ***p* < 0.001**, d.f. = 5168	*r* = −0.02 (−0.16, 0.13) *p* = 0.84, d.f. = 172
presence/absence of fire for fuel management (state/NGO)	*η*^2^ = 0.10 (0.03, 0.14) ***p* < 0.001**, d.f. = 7236	*η*^2^ = 0.17 (0.07, 0.23) ***p* < 0.001**, d.f. = 5169	*r* = 0.02 (−0.13, 0.17) *p* = 0.786, d.f. = 173
respondents’ perception of policy effectiveness	*η*^2^ = 0.04 (0.02, 0.049) ***p* < 0.001**, d.f. = 7 2170	*η*^2^ = 0.07 (0.05, 0.10) ***p* < 0.001**, d.f. = 5 1080	*r* = −0.22 (−0.27, −0.16) ***p* < 0.001**, d.f. = 1085

### (b) Distribution of human fire use

**Figure 1.**
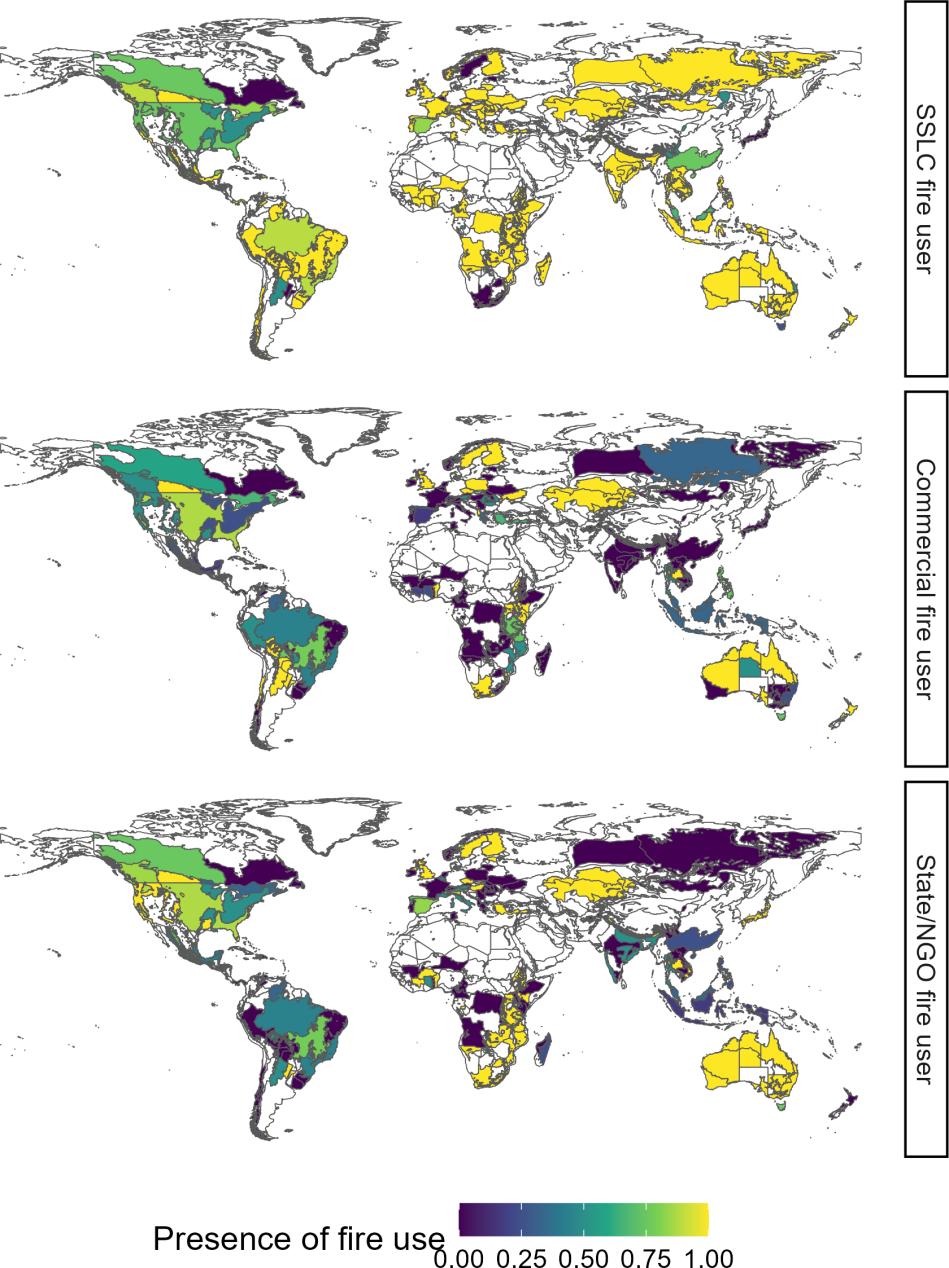
Global distribution of fire use, grouped by fire user type. Human fire is widespread and primarily used by small-scale livelihood and/or cultural fire users. State/NGO fire use is the most spatially heterogeneous, with apparently widespread use in Australia and parts of southern Africa but with seemingly less uptake in South America. A value of 0 denotes that all respondents agreed the fire user types to be absent, while 1 indicates agreement between respondents that the fire user type is present. Values between 0 and 1 indicate that survey respondents disagreed on whether the fire user type is present (i.e. 0.5 indicates half believed it was present, half absent).

Survey responses demonstrate that human fire use is extremely widespread and diverse. SSLC fire users are the most widespread globally (figure 1). SSLC fire use was deemed present in 88% of regions with a survey response, compared with 40% for commercial fire use and 53% for state/NGO fire use (note that for regions with multiple respondents, we count something as present if over half of respondents deemed it so) . The presence of state/NGO fire use varies most across continents, ranging from 29% in South America to 83% in Oceania. Neither the presence of commercial fire use or SSLC fire use varies significantly across continents (table 2). SSLC fire use ranges from 82% presence in Asia to 96% presence in South America, while commercial fire use ranges from 23% presence in Asia to 59% presence in North America.

In line with continent-level variation, there are no significant differences between responses based on respondents’ backgrounds for SSLC fire use and commercial fire use, but there are differences in responses for state/NGO fire use (table 2). While researchers thought state/NGO fire use present in 42% of regions, this rose to 70% for government employees and 86% for employees of companies using fire. Hence, the presence of state/NGO fire use is the most variable geographically, and the fire use context that generates the most contrasting responses between respondents is based on their sectors.

Modal fire uses vary across fire user types. For state/NGO fire users, the most common fire use purposes (present in 28–37% of survey regions with responses; others ≤ 11%) are to manage fuel loads, create fire breaks or backburning to combat an active wildfire. Fire use for fuel load reduction varies significantly by continent but interestingly has no relationship with GFED5 burned area (*r* = 0.02; table 2). SSLC fire use is the most diverse, with seven fire use purposes occurring more frequently than the most frequent fire use purpose by another user group (i.e. >37% of regions with state/NGO use of prescribed fire). Of these, the most frequent purposes are the initial clearance of vegetation to establish permanent agriculture (present in 71% of regions with responses) and clearance of land for swidden or semi-permanent agriculture (67%). The second most common reason for burning is to clear vegetation to establish pasture (58%), followed by burning to create habitat for hunting or fishing (41%) and combat pests and parasites (39%). For commercial fire users, the top five fire uses (spanning 16–24% of regions with responses) all relate to agriculture (including vegetation clearance and crop residue burning between subsequent crops) and pasture management.

Reasons for burning are relatively consistent across continents for each fire user type. In line with the global trend, in five of six continents, the most frequent reason for state/NGO fire use is to reduce fuel-loads to reduce risk of wildfires, while in Africa the most frequent reason for state/NGO fire use is ecosystem management for nature conservation. Similarly, in five continents, the most frequent reason for commercial fire use is either to clear vegetation for agriculture or remove agricultural residues. South America is the exception, where pasture maintenance is the most frequent reason for burning (present in 38% of regions with a response). Similarly, across all continents, the most frequent reasons for SSLC fire use are either to clear vegetation for swidden, semi-permanent or permanent agriculture. Vegetation clearance for swidden or semi-permanent agriculture was the most frequently reported reason for regions in Asia, South America and Africa, with vegetation clearance to establish permanent agriculture most frequent elsewhere. Besides these two apparently dominant reasons for burning, burning to create firebreaks is most common in Oceania (reported in 78% of regions with responses), burning to maintain pasture most frequent in Europe (62%), burning to clear vegetation to establish pasture is most common in South America (79%) and North America (64%) outcomes such as biodiversity conservation and burning to remove weeds and crop residues most frequent in Africa (76%) and Asia (54%).

### (c) Distribution and perceptions of policy interventions targeting fire use

**Figure 2.**
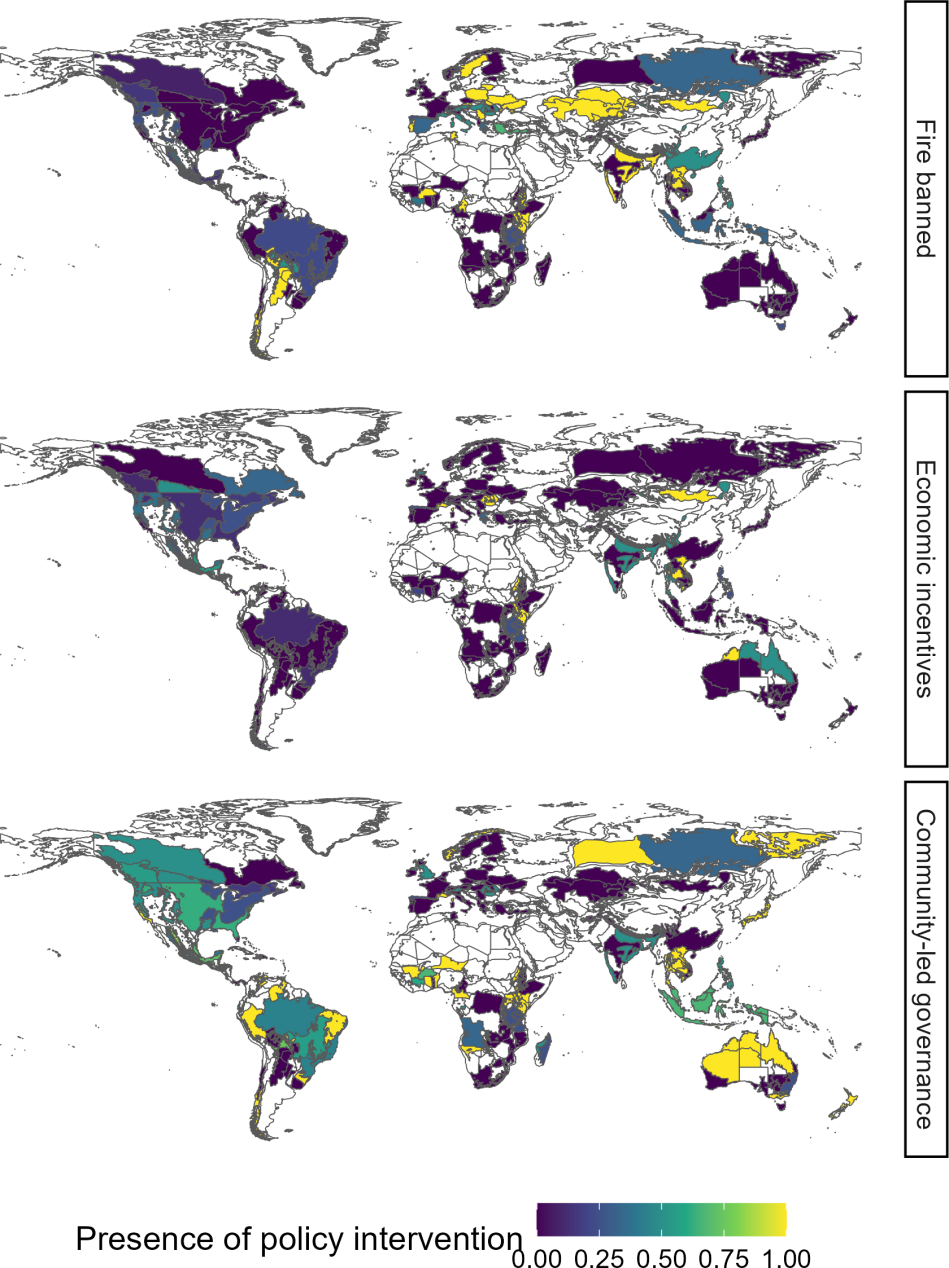
Distribution of selected policy types, demonstrating contrasting approaches to fire policy—outright bans on fire use (option a in table 1), economic incentives to reduce fire use (option e) and community-led governance of fire (option g). A value of 0 denotes that all respondents agreed the policy intervention not to be present, while 1 indicates agreement between respondents that the policy intervention is present. Values between 0 and 1 indicate disagreement between survey respondents, perhaps indicating that policy only applies in restricted areas or has limited impact or enforcement.

Globally, the survey shows that the most frequent policy approaches to human fire use are to legislate to ban burning fully or partially (response options a, b and c in table 1), or otherwise to restrict it (option d) (figure 2; see electronic supplementary material S2 and figure S2.2a,b for maps of distribution of all types of policy intervention). At least one form of ban or regulation (options a, b, c and d) exists in 87% of regions with a survey response. Multiple forms of ban or regulation are present in 37% of regions. Another common policy response to human fire use is educational campaigns to restrict fire use (option f), which exist in 58% of regions. In contrast, survey respondents only deemed community-led fire governance (option g) to be present in 34% of regions and economic incentives to limit fire use (option e) in 9%.

There are weakly significant differences in fire policies in force across continents (table 2). In four out of six continents, the most common form of policy intervention is a restriction short of an outright ban; in Africa (55% of regions with a response) the restrictions are most commonly on fire use in protected areas (response option c), while in Europe (67%), North America (87%) and Oceania (91%) restrictions most commonly make fire use conditional e.g. on having a permit (response option d). In contrast, in Asia (64% of regions with a response) and South America (70%), educational campaigns are the most common type of policy intervention. However, in both Asia and South America, the second most common form of policy intervention is also a restriction short of an outright ban (response options b, c or d).

**Figure 3.**
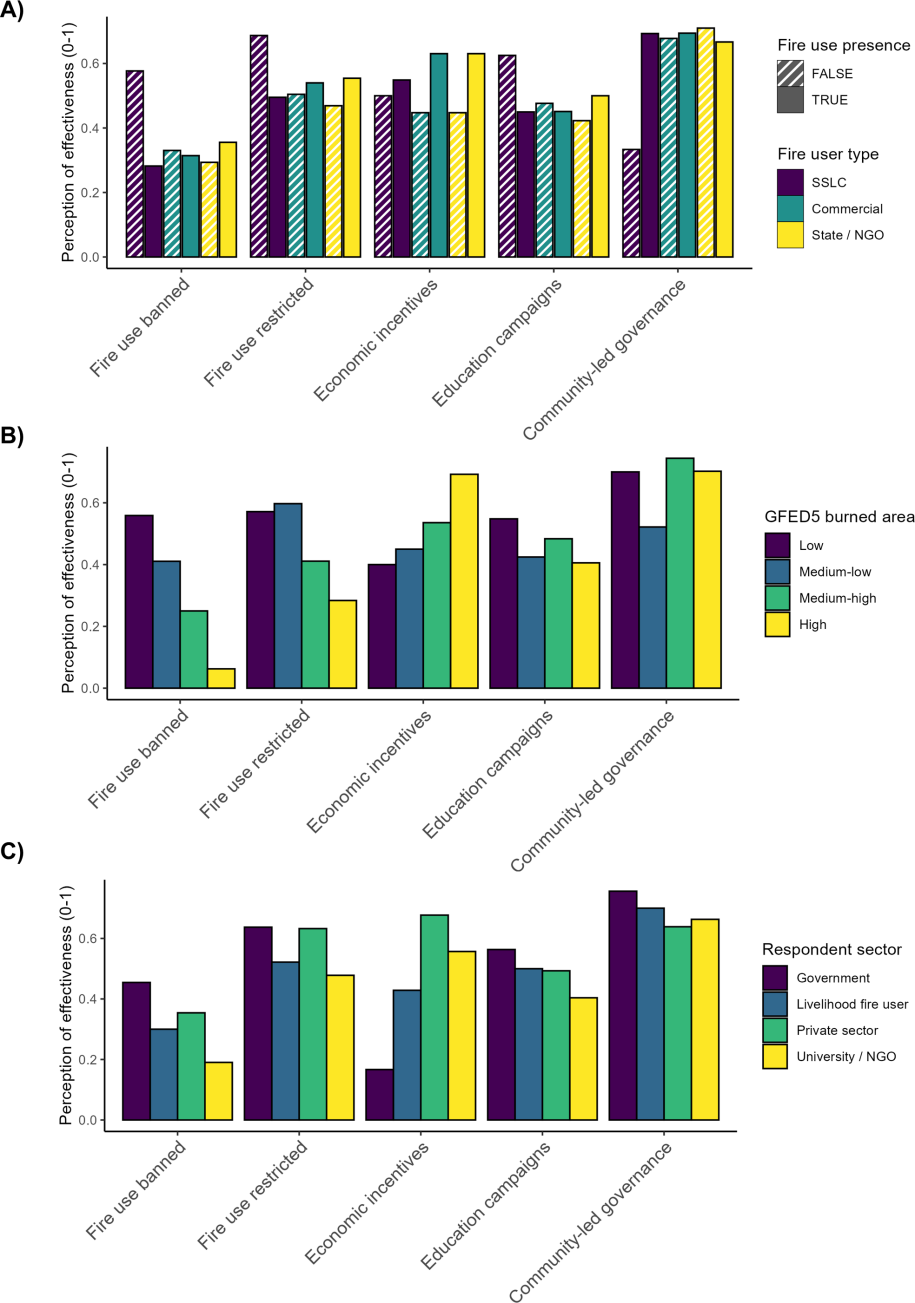
Distribution of policy intervention types and perceptions of their effectiveness. (A) Policy effectiveness given the presence/absence of fire use by different fire user types; (B) Survey respondents’ perceptions of effectiveness of policy intervention types across quartiles of GFED5 burned area and (C) respondents’ perceptions of effectiveness of policy intervention types based on their sectors. GFED5 = 5th Global Fire Emissions Database. Fire use restricted is the mean of values for options b–d in table 1, which cover different forms of fire-use restriction.

While community-led governance is comparatively rare, survey respondents deemed it the most effective form of governance for their respective regions. Survey respondents rated it at 67% of the maximum possible effectiveness, compared with just 32% for all-out fire bans (response option a), the least effective policy intervention type in survey responses. Policy intervention effectiveness varies markedly with burned area (figure 3B). In the lowest quartile of GFED5 burned area, policy intervention effectiveness averages 64% of the maximum possible and is lowest at 40% in the highest quartile of GFED5 burned area (correlation test: *r* = −0.22; table 2). Indeed, in the lowest quartile of burned area, fire bans (option a) were deemed somewhat effective—64%—but were deemed to have an effectiveness of only 3% in the highest burned area quartile. In contrast, community-led governance was believed to be consistently effective across regions regardless of burned area, from 69% in the upper quartile of GFED5 burned area to 75% in the lowest quartile. Similarly, economic incentives seem to grow in effectiveness with increased burned area, though with a small sample size (*n* ≤ 25 by GFED quartile). Partial bans and restrictions (options b, c and d) follow an intermediate course, ranging from 57% in the lowest quartile of GFED5 burned area to 33% in the highest quartile.

There is an apparent tension between SSLC fire use and fire policy (figure 3A). SSLC fire use is associated (perhaps tautologously) with decreased effectiveness of fire bans (response option a, b and c) (decrease of 30%), fire use restrictions short of a total ban (option d) (19% decrease), and education campaigns (18% decrease). In contrast, SSLC fire use is associated with the increased effectiveness of community-led fire governance (36% increase). SSLC fire use is also more frequently present where community-led governance is in place (97% of cases) compared with 79% of cases where community-led governance is absent. Combined with the changes in effectiveness across GFED5 burned area, we may conclude that the effectiveness of different fire policy measures is heterogeneous, with outright bans on fire use (option a), for example, unsuited to areas where biophysical and socio-economic conditions make fire an intrinsic component of ecosystems and/or livelihoods (figure 3B).

Finally, there are smaller, but significant, effects in the relationship between survey respondents’ views of policy intervention effectiveness and respondents’ backgrounds (figure 3C; table 2). Government respondents were most favourable about fire bans (response option a), while private sector respondents were most favourable towards economic incentives, and university and NGO respondents most favourable towards community-led governance.

## 4. Discussion

We presented data from a global survey of experts in human–fire interactions, contributing to the synthesis of knowledge of human fire use. Survey responses demonstrate that human fire use is very widespread globally (figure 1). Human fire is present in 97% of regions with a survey response, with multiple of our fire user categories present in 47%. Reasons for fire use are also extremely diverse. All 29 fire use purpose categories included in the survey exist in at least one region. These findings reinforce literature suggesting human fire use plays an important role in global fire regimes; one that has been underappreciated by the global scientific community and policymakers [19,34].

Data in the GFUS suggest that fire use associated with small-scale livelihoods and cultural reasons is widespread globally, and more so than burning by state agencies, NGOs and commercial enterprises. While ecological outcomes, wildfire risk and greenhouse gas emissions are usually central in global fire assessments, the GFUS makes clear that policymakers cannot ignore the economic and cultural benefits of fire use to rural populations globally. The survey highlights the challenge that policymakers face worldwide, in equitably balancing the needs of rural fire users with considerations such as climate change or biodiversity conservation. Research must continue to interrogate whether and how these outcomes trade off with one another in specific contexts. In the GFUS, vegetation clearance to establish permanent agriculture is the most reported reason for burning by both SSLC and commercial fire users across the surveyed regions, suggesting a particular need to understand the outcomes of fire associated with agricultural expansion. Global syntheses highlight how wider economic, agricultural and land policies are driving changes in rural fire use in many regions [1,35]. The GFUS data suggest that restrictive fire policies are ineffective in many regions, supporting the assertion that fire policy should not be designed in isolation, without considering wider structural conditions that shape fire use, such as land reform or economic drivers towards agricultural intensification.

Prescribed burning to reduce fuel loads is the most common reason for burning among state/NGO fire users, but this is only present in 37% of regions with a survey response, varies significantly across continents, and shows no correlation with burned area. Thus, state/NGO adoption of burning to manage fuel loads appears linked to heterogeneity in local institutional and socio-ecological contexts at a finer scale than captured in this survey. Future research should further interrogate these factors, synthesizing knowledge about the conditions under which controlled burning should be promoted as a management tool for reducing the severity of wildfires and restoring fire-dependent ecosystems, and where other fire management tools may be more appropriate.

The survey data imply that governance approaches may be poorly suited to fire regimes resulting from climate and land use change [36,37]. Notably, the most frequent policy interventions are also those that lose their effectiveness with increasing burned area (figure 3B). Community-led fire governance meanwhile, which is comparatively rare, does not appear to lose effectiveness with increasing burned area (figure 3B) and achieves the highest mean effectiveness rating from survey participants (67%, 59% and others). This suggests a need for future research to synthesize knowledge about how communities govern fire use locally, including through more tacit rules and norms that structure fire’s application to the landscape. There is also a need to better understand factors (e.g. security of land tenure) that strengthen community fire governance. The GFUS did not ask respondents about state and NGO policies supporting community-led governance, and this is also an important area for future study.

More broadly, the survey data meaningfully expand global data coverage on human–fire interactions: 22% of survey regions with a response have no data in the DAFI or LIFE (electronic supplementary material, S3). Where comparison was possible with these databases, there was generally moderate agreement (electronic supplementary material, S3), indicating that such data might be carefully combined towards increasingly complete global data coverage. Furthermore, the GFUS dataset also includes substantial contributions from fire practitioners whose experiential knowledge has been largely overlooked in highly cited fire literature [15]. Strong correlation between levels of agreement among multiple respondents answering for the same survey regions and their confidence in their answers is also a positive indicator of the value of the GFUS data (electronic supplementary material, S3).

Yet, the GFUS dataset is also strongly limited in its geographical scope and in the voices it represents. Survey responses clustered in regions of the Global North with significant fire management challenges [38,39] (electronic supplementary material, S2 and figure S2.1), but regions of the world with the most burned area, particularly in sub-Saharan Africa, were underrepresented. Researchers dominated among respondents, and those fire practitioners that responded tended to work for state agencies or companies, rather than using fire within small-scale livelihoods or for cultural reasons. Indeed, the dataset underscores that ways of experiencing, understanding and knowing fire regimes are fragmented across different sectors and contexts, suggesting need for better communication and collaboration in management and governance of fire [8,23,24]. For example, where there were multiple survey respondents for a given region, there was relatively high disagreement over presence/absence of different policy intervention types (entropy of 0.48 versus 0.37 overall; see electronic supplementary material, S3). Furthermore, understanding of policy intervention effectiveness differed significantly—and perhaps stereotypically—across survey respondents’ sectors (figure 3C). Further collaboration to produce and analyse datasets like the GFUS data may be a productive way to generate dialogue and shared knowledge between different kinds of fire experts. Inclusion of more diverse voices in such efforts, with the aim of identifying equitable fire governance approaches, would be highly valuable, but must be facilitated carefully to ensure that participation is not extractive.

## 5. Conclusion

The GFUS was the first global effort to elicit expert knowledge about contemporary human fire use and policy interventions affecting it. It contributes to global knowledge, while highlighting important geographical data gaps in areas with high burned area. The data demonstrate that fire use is generally dominated by people using fire to meet small-scale livelihood objectives and/or for cultural reasons, with state, NGO and commercial fire use more limited geographically. The data point to agriculture as a dominant reason for human fire use, highlighting a need to better understand variation in agricultural fire use and its outcomes.

Survey respondents deem community-led fire governance more effective than state regulations and communication campaigns, especially in more flammable regions. Yet, the distribution of community-led fire governance is patchy. Policymakers must better understand these local forms of fire governance, and whether and how they can be supported by external policies. In this study, we only assessed the effectiveness of policy interventions vis-a-vis their own aims regarding fire use. There is a need for future research to synthesize global knowledge about fire policy effectiveness in terms of broader outcomes related, for example, to wildfire risk reduction, biodiversity or livelihoods. The GFUS highlights many ways that societies benefit from controlled fire use, from individual households with land-based livelihoods to whole communities at risk of wildfire. What will be vitally important going forward is understanding better how the economic and cultural benefits of fire use trade off against or align with other outcomes such as biodiversity conservation or climate change mitigation, and how equitably the outcomes of fire use and fire policy are distributed.

## Data Availability

The data from the global fire use survey, alongside code to process it and reproduce figures and statistical tests presented here are made freely available online via Zenodo [40]. The survey data are also available in an interactive web application at: https://olperkins1987.shinyapps.io/Global-Fire-Use-Survey/. Supplementary material is available online [41].
